# Case series: Reinfection of recovered SARS CoV‐2 patients for the third time

**DOI:** 10.1002/ccr3.4936

**Published:** 2021-10-15

**Authors:** Sepideh Hasanzadeh, Somayeh Sadat Shariatmaghani, Atefeh Vakilian, Alireza Javan, Mahsa Rahmani, Sara Ganjloo, Mahbubeh Jangi, Saeid Amel Jamehdar

**Affiliations:** ^1^ Antimicrobial Resistance Research Centre Mashhad University of Medical Sciences Mashhad Iran; ^2^ Department of Microbiology and virology School of Medicine Mashhad University of Medical Sciences Mashhad Iran; ^3^ Department of Internal Medicine School of Medicine Mashhad University of Medical Sciences Mashhad Iran; ^4^ Department of Infectious Diseases School of Medicine Mashhad University of Medical Sciences Mashhad Iran; ^5^ Faculty of Veterinary Medicine Ferdowsi University of Mashhad Mashhad Iran

**Keywords:** clinical case series, reinfection, SARS‐CoV‐2

## Abstract

This set of cases provides important evidence of re‐infection and recurrence of SARS‐CoV‐2 even for the third time. Consequently, this possibility should be considered more in recurrent patients with Covid‐19 symptoms.

## Graphical abstract

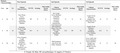

## 1 INTRODUCTION

The outbreak of coronavirus disease 2019 (COVID‐19) rapidly spread around the world. Several studies on re‐positive cases after complete recovery caught our attention. Here, we reported the re‐positive RT‐qPCR for severe acute respiratory syndrome coronavirus 2 (SARS‐CoV‐2) after complete recovery. Our results are just a report and need to be confirmed by a larger cohort.

Less than a year ago, coronavirus disease 2019 (COVID‐19) was discovered in a Chinese province, spread rapidly across continents, which generating a pandemic with serious consequences for the health of the world.^1^ As of December 8, 2020, Covid‐19 had more than 67.6 M confirmed cases, with more than 1.54 M deaths around the world.^2^ COVID‐19 has had a wide range of symptoms which include: fever, breathlessness, cough, sore throat, headache, muscle pain, chills, and loss of taste or smell.^3^

Most people who get COVID‐19 have mild (with no sign of pneumonia) or moderate symptoms (including fever and respiratory symptoms with radiological findings of pneumonia), then severe symptom (dyspnea, respiratory frequency ≥30/min, and blood oxygen saturation ≤93%), and finally critical symptom (respiratory failure, septic shock, and/or multiple organ dysfunction/failure).^4^, ^5^

Most recent studies show that immunity after acute respiratory syndrome infection can develop in infected people.^6^ This immunity is not permanent and creates a sense of false protection for people who have already been infected and defeated the disease.^7^ The present report which was conducted at the Mashhad University of Medical Sciences described the possibility of SARS‐CoV‐2 re‐infection even for the third time.

### 1.1 Case description

Clarifying the characteristics and frequency of re‐infection is important because it can affect our understanding of acquired immunity after natural infection. A COVID‐19 positive was defined by two criteria:

- Show at least one clinical sign of COVID‐19 including flu‐like symptoms such as fever, chills, shortness of breath, anosmia, or dyspepsia
- a positive SARS‐CoV‐2 RT‐PCR test result.

Here, we present three re‐infected cases who went to the laboratory for re‐testing, whose clinical and laboratory characteristics are shown in Table 1.

**TABLE 1:** Clinical characteristics of COVID‐19 first, second, and third episodes.

Patients Characteristics			First Episode				2nd Episode				3rd Episode			
Case	Age	Sex	Clinical Characteristics	RT PCR	Serology	Intervening Period (Days)	Clinical Characteristics	RT PCR	Serology	Intervening Period (Days)	Clinical Characteristics	RT PCR	Serology	Intervening Period (Days)
1	44	F	Sore Throat, Fever, Headache, Dyspnea, Tachycardia Diarrhea	NP	N	21	Dry Cough, Fever, Shortness Of Breath, Weakness, Excessive Sweating, Hypotension, Acute Depression	21	N	18	Cough, Severe Weakness, Myalgia And Bone Pain, Fever, Mild Chills	16	N	24
2	40	M	Headache, Fever, Cough, Chills, Proteinuria, Diarrhea	30.3	N	17	Mild Headache, Runny Nose And Body Aches, Cough, Paresthesia, Fatigue	32.6	NP	10	Cough, Severe Weakness, Myalgia, Paresthesia, Fever, Diarrhea, Nausea	24.3	N	15
3	50	M	Fever, Dyspnea, Cough, Headache, Dizziness, Weight Loss, Severe Weakness, Loss Of Appetite.	12.8	P	23	Mild Symptoms Like Fever And Chills, Weakness	12.8	P	15	Fever, Chills And Severe Weakness, Severe Diarrhea, Weight Loss, Shortness Of Breath, Loss Of Appetite	21	P	28

Abbreviations: F, Female; M, Male; N, negative; NP, not performance; P, Positive.

## 2 CASE 1

A 58‐year‐old female, with no underlying disease just occasionally mild flu‐like symptoms (sore throat, fever, headache, dyspnea, tachycardia, and diarrhea) was referred on March 18, 2020. The first chest x‐ray revealed ground‐glass opacity At this stage, the diagnosis is made solely on the basis of clinical signs and was subjected to treatment with chloroquine and Oseltamivir.

Blood test results were normal regarding both inflammation and respiratory parameters. Qualitative IgG/IgM was negative for both antibodies at the end of the symptoms. The patient was isolated at home and three weeks later the SARS‐CoV‐2 PCR assay was negative.

Approximately 93 days after her initial symptoms, she presented new clinical symptoms with dry cough, fever, shortness of breath, weakness, excessive sweating, hypotension, and acute depression. Resting heart rate (75/min) and oxygen saturation (less than 85%) and other symptoms led to hospitalization. During the hospitalization, blood test results showed leukocytosis 13000 with lymphopenia, increased D‐dimer (240 ng/ml), and ferritin (267 mg/ml). On May 26, 2020, the SARS‐CoV2 RT‐PCR assay was positive with a Ct value of 21 and qualitative IgG/IgM was negative for both antibodies at the end of symptom. About 65 days later, recurrent symptoms of cough, severe weakness, myalgia and bone pain, fever, and mild chills appeared. The SARS‐CoV2 RT‐PCR assay was positive, but a CT scan was not performed. Cough and chest pain lasted up to two months. The cough has continued and led to the use of various inhalation sprays.

## 3 CASE 2

A 48‐year‐old male was presented with headache, fever, cough, chills, Proteinuria, diarrhea on March 9, 2020. A nasopharyngeal swab was positive for SARS‐CoV‐2 with a Ct value of 30.3. The patient was self‐isolated at home and most symptoms vanished after a few days except coughing. Four months (July 13, 2020) later, the patient complained of a mild headache for three nights. More symptoms appeared with a runny nose and body aches, cough, paresthesia, and fatigue. The symptoms lasted for 10 days and were resolved without hospitalization. His nasopharyngeal swab was again positive for SARS‐CoV‐2 (Ct value 32.6). Chest X‐ray was not performed and complete blood count was normal.

After 98 days, in November 2020, symptoms like cough, severe weakness, myalgia, paresthesia, fever, diarrhea, and nausea reappeared. There were no severe respiratory symptoms but mild fever and gastrointestinal symptoms. The SARS‐CoV‐2 PCR assay was positive (Ct=24.3). The resting heart rate was 96/min with an oxygen saturation of 94%. In the first period of infection with Covid‐19, his heart rate was consistently 94–96/min, whereas, during the second time, it was consistently 92–99/min. During all periods, viral serology revealed negative SARS‐Covid‐2 IgM and IgG antibodies.

## 4 CASE 3

In April 2020, a 46‐year‐old male presented with three days of intense headache and drowsiness. After four days, he complained about fever, shortness of breath, cough, headache, dizziness, weight loss, severe weakness, and loss of appetite. His pulse was 95/min during rest with oxygen saturation of 89% and a rapid antibody test was positive for IgM and IgG. The RT‐PCR test was positive (Ct=12.85). Subsequently, the patient's symptoms improved, and after three weeks, the SARS‐CoV‐2 PCR assay was negative. In July 2020, the patient presented again with mild symptoms like fever and chills, weakness. The SARS‐CoV‐2 PCR test showed a positive result (24.3) and oxygen saturation was 90%. Despite moderate symptoms and dyspnea, the patient was not hospitalized and his clinical condition improved after 15 days.

Four months later, the symptoms were more severe including fever (>40 degrees), chills and severe weakness, severe diarrhea, weight loss, shortness of breath, and loss of appetite. The SARS‐CoV‐2 PCR assay was positive with a Ct value of 21. Finally, a quantitative antibody test showed positive for both IgG and IgM antibodies.

## 5 DISCUSSION

About 12 months after the emergence of the first cases of COVID19, there is still clinical suspicion of reinfection by SARS‐CoV‐2, although some reports have been evaluated by different scientists globally.^7^, ^8^, ^9^ The clinical sign of reinfections is frequently milder than those of the primary infections.^10^

In our study, the clinical manifestations of reinfection differed across all three cases: one person showed mild symptoms, two showed moderate symptoms, required oxygen support. Serological test results were variable in all patients over three periods.

According to the World Health Organization, there is no sufficient evidence to verify antibody‐mediated immunity in this epidemic to ensure the safety of the individuals.^11^ The re‐positive RT‐qPCR assays of SARS‐CoV‐2 after complete recovery and negative results might be attributed to various hypotheses like reactivation of the virus, persistent infection, new infection with the same or different virus, and laboratory errors strain.^12^

Two of these are more likely: One of them is the possibility of viral reactivation which is related to the host immunity status, virological factors, and type and immunosuppression level.^13^ Another one is the possibility of false‐negatives result in SARS‐CoV‐2 PCR assays in those improved patients due to limitations of test methods.^14^ In addition, it is not obvious whether the immune response is protective in every infected patient and how long its’ protective effect lasts. Likewise, animal studies show a temporary immune response against re‐infection.^15^

In our cases, patients who recovered at least two months from COVID‐19, later developed Covid‐19 for the third time. Due to high suspicion of COVID‐19 based on clinical signs, nasopharyngeal swabs were sent for RT‐PCR, which were positive. Unfortunately, because of the technical limitations of the SARS‐CoV‐2 serological tests, variable results were obtained in all of our three cases.

## 6 CONCLUSION

This set of cases provides important evidence of re‐infection and recurrence of SARS‐CoV‐2 even for the third time. Consequently, this possibility should be considered more in recurrent patients with Covid‐19 symptoms.

## CONFLICTS OF INTEREST

The authors declare that there is no conflict of interest.

## AUTHOR CONTRIBUTIONS

Sepideh Hasanzadeh and Saeid Amel Jamehdar designed the work and interpreted the data. Sara Ganjloo, Mahsa Rahmani, and Mahbubeh Jangi revised the manuscript critically for important content. Somayeh Sadat Shariatmaghani, Atefeh Vakilian, and Alireza Javan critically revised the article for important intellectual content. All authors reviewed and approved the final version of the manuscript.

## CONSENT

Appropriately written informed consent was obtained for publication of this case report and accompanying images.

## Data Availability

Data presented in this manuscript are available upon request.
